# A Novel Electrophototrophic Bacterium *Rhodopseudomonas palustris* Strain RP2, Exhibits Hydrocarbonoclastic Potential in Anaerobic Environments

**DOI:** 10.3389/fmicb.2016.01071

**Published:** 2016-07-12

**Authors:** Krishnaveni Venkidusamy, Mallavarapu Megharaj

**Affiliations:** ^1^Centre for Environmental Risk Assessment and Remediation, University of South Australia, Mawson Lakes, SAAustralia; ^2^CRC for Contamination Assessment and Remediation of the Environment, Mawson Lakes, SAAustralia; ^3^Global Centre for Environmental Risk Assessment and Remediation, The University of Newcastle, Callaghan, NSWAustralia

**Keywords:** microbial electrochemical remediation, hydrocarbonoclastic bacterium, photoorganotrophic hydrocarbon degradation, electrode respiring bacteria, *Rhodopseudomonas palustris* strain RP2

## Abstract

An electrophototrophic, hydrocarbonoclastic bacterium *Rhodopseudomonas palustris* stain RP2 was isolated from the anodic biofilms of hydrocarbon fed microbial electrochemical remediation systems (MERS). Salient properties of the strain RP2 were direct electrode respiration, dissimilatory metal oxide reduction, spore formation, anaerobic nitrate reduction, free living diazotrophy and the ability to degrade n-alkane components of petroleum hydrocarbons (PH) in anoxic, photic environments. In acetate fed microbial electrochemical cells, a maximum current density of 305 ± 10 mA/m^2^ (1000Ω) was generated (power density 131.65 ± 10 mW/m^2^) by strain RP2 with a coulombic efficiency of 46.7 ± 1.3%. Cyclic voltammetry studies showed that anaerobically grown cells of strain RP2 is electrochemically active and likely to transfer electrons extracellularly to solid electron acceptors through membrane bound compounds, however, aerobically grown cells lacked the electrochemical activity. The ability of strain RP2 to produce current (maximum current density 21 ± 3 mA/m^2^; power density 720 ± 7 μW/m^2^, 1000 Ω) using PH as a sole energy source was also examined using an initial concentration of 800 mg l^-1^ of diesel range hydrocarbons (C9-C36) with a concomitant removal of 47.4 ± 2.7% hydrocarbons in MERS. Here, we also report the first study that shows an initial evidence for the existence of a hydrocarbonoclastic behavior in the strain RP2 when grown in different electron accepting and illuminated conditions (anaerobic and MERS degradation). Such observations reveal the importance of photoorganotrophic growth in the utilization of hydrocarbons from contaminated environments. Identification of such novel petrochemical hydrocarbon degrading electricigens, not only expands the knowledge on the range of bacteria known for the hydrocarbon bioremediation but also shows a biotechnological potential that goes well beyond its applications to MERS.

## Introduction

Soil and groundwater petroleum hydrocarbon contamination has long been a serious concern to environmental and public health. Of these petroleum hydrocarbon (PH) contaminants, diesel range hydrocarbons (DRH) have been documented as one of the most abundant pollutants; they can be biodegradable in both oxic and anoxic conditions (Huang et al., 2011). Microbial remediation of these petrochemical compounds is claimed to be an efficient, economic, versatile alternative to the physicochemical methods. However, the rate of microbial utilization of these PH compounds is very slow especially in anaerobic environments where the availability of relevant electron acceptors is limited. The recent research on such recalcitrant contaminant removal using bioelectrochemical systems is leading to a new interest in its practical applications. An emerging microbial electrochemical remediation systems (MERS) shows potential as an effective approach that can exploit microorganisms for treating contaminants while generating electricity in the process. This has recently been proposed for the remediation of PH contaminants by capitalizing on the bio-catalytic potential of electrode respiring bacteria (ERB; Morris et al., 2009; Venkidusamy et al., 2016).

Electrode respiring bacteria are a group that has received much attention in the field of electromicrobiology because of their exoelectrogenic capabilities to degrade substrates that range from easily degradable natural organic compounds to xenobiotic compounds such as PH contaminants. Many studies have shown the presence of diverse, electro active-microbial communities on fuel cell electrodes including members of the *Alphaproteobacteria* (Zuo et al., 2008), *Betaproteobacteria* (Chaudhuri and Lovley, 2003), *Gammaproteobacteria* (Kim et al., 2002), *Deltaproteobacteria* (Holmes et al., 2006), and *Firmicutes* (Wrighton et al., 2008). Of these, *Gammaproteobacteria* was the dominant class found on the anodes of organic contaminants such as PH fed MERS (Morris et al., 2009; Venkidusamy et al., 2016). Several bacterial strains from this class have been isolated either from electrochemical systems fed with wastewater or defined carbons sources and their physiological roles have been studied. However, the microbial community composition is divergent in MERS (Morris et al., 2009; Venkidusamy et al., 2016), and the physiology of such populations remains to be investigated. Therefore, identification of such ERB which can be used in MERS systems for the enhanced removal of PH contaminants is of current importance.

In the present study, such a novel electrode respiring, hydrocarbonoclastic bacterial strain was isolated from the biofilms of PH fed MERS anodes and analyzed its physiology and activities in pure culture studies. This strain was found to be a Fe (III) respiring bacterium, phylogenetically related to *Rhodopseudomonas palustris* and designated as *R. palustris* strain RP2. The electrochemical activity was determined by using cyclic voltammetry and fuel cell techniques. Here, we show the existence of hydrocarbonoclastic behavior by the strain RP2, first such organism from MERS environments with several novel features.

## Materials and Methods

### Bacterial Strain

The bacterium used in this study was isolated from the anodic biofilm of a MERS through serial dilution techniques. The initial source of inoculum for the PH fed MERS was a PH contaminated ground water inoculated with activated sludge. Bacterial cells of the anodic biofilm were extracted into a sterile phosphate buffer and shaken vigorously to separate cells from the electrode. The extracted cell suspensions were serially diluted and plated on modified Hungate’s medium (Hungate, 1950) and incubated anaerobically in a glove box (Don Whitley Scientific, MG500, Australia) for a period of two weeks. Single colonies were randomly selected and transferred to anaerobic agar plates filled with Luria Bertani (LB) medium (Sambrook et al., 1989). Cultures were routinely cultivated using anoxic rich medium (LB) under illuminated conditions. A chemically defined medium supplemented with Wolfe’s trace elements and vitamins was used in the fuel cell experiments as previously described (Oh et al., 2004).

### Culture Conditions and Biodegradation Experiments

Bacterial cells were grown under anoxic photosynthetic conditions with acetate (20 mM) as an electron donor in phosphate buffer saline supplemented with Wolfe’s trace elements and vitamins and sealed with aluminum crimps. For experiments with different electron acceptors 10 mM nitrate, 10 mM sulfate, 10 mM iron (III) citrate, and 10 mM iron (III) oxide were used. The cells were cultured under different physiological conditions, including phototrophic and chemotrophic, oxic and anoxic conditions. The photosynthetic pigments were analyzed by measuring the whole cell absorption spectra in the UV-visible ranges from 300 to 1100 nm as described by Mehrabi et al. (2001). Biolog-GN2 plates were used to determine the carbon source utilization using the strain RP2 (Biolog., USA) according to the manufacturer’s instructions under anoxic, illuminated conditions. The cells were also tested for their growth with nitrogen-deficient solid medium lacking a carbon source according to Kranz and Haselkorn (1986). DRHs was also used as the electron donor in biodegradation experiments at a concentration of 4000 mg l^-1^. Hydrocarbonoclastic potential of the strain RP2 was monitored under different electron accepting, growth environments (nitrate, sulfate, iron (III) as terminal electron acceptors) including phototrophic and chemotrophic conditions. All cell cultures were maintained in triplicates for each experiment. All procedures for anoxic growth experiments, from medium preparation to manipulating the strain were performed using standard anoxic conditions. All culturing was prepared in sealed serum vials with nitrogen/carbon dioxide (80:20, v/v) in the headspace.

### Fe(III) Oxide Reduction

For investigating Fe(III) respiration process, cells were grown in two different environments: anoxic photoheterotrophic and chemoheterotrophic culture conditions supplemented with crystalline Fe(III) oxide (10 mM) as the terminal electron acceptor. The cells were grown in Wolfe’s medium using acetate (20 mM) supplemented with trace elements and vitamins (Lovley and Phillips, 1988a). Fe(III) reduction was determined using the ferrozine assay (Lovley and Phillips, 1988b). The bacterial suspension was added to a pre-weighed vial containing 0.5 M HCL. HCL extracted samples were added to 5 ml of ferrozine (1 g l^-1^) in 50 mM HEPS buffer. The filtered samples were then analyzed in a UV-Vis spectrophotometer (maxima@λ 562 nm) to quantify the Fe(II) formation as previously described (Lovley and Phillips, 1988b).

### Microscopy

Samples for transmission electron microscopy were fixed in electron microscopy fixative (4% paraformaldehyde/1.25% glutaraldehyde in PBS, + 4% sucrose, pH-7.2) and washed with buffer. Samples were postfixed in 2% aqueous osmium tetroxide. They were dehydrated in a graded series of ethanol, and then infiltrated with procure/araldite epoxy resin. Blocks were polymerized overnight at 70°C. Sections were cut on a Leica UC6 Ultramicrotome using a diamond knife, stained with uranyl acetate and lead citrate, and examined in a FEI Tecnai G2 Spirit Transmission Electron Microscope. The electrode samples were fixed and prepared as described earlier (Venkidusamy et al., 2016). The dried brush samples were examined using a scanning electron microscope (Quanta FEG 450, FEI) at an accelerating voltage of 20 kV.

### 16S rRNA Gene and Phylogenetic Analysis

Genomic DNA of *R. palustris* strain RP2 was extracted from photoheterotrophically grown cells by using the UltraClean microbial DNA isolation kit (MO BIO, Carlsbad, CA, USA) following the manufacturer’s instructions. The universal primers were used to amplify16S rRNA gene according to the procedure devised by Weisburg et al. (1991). The PCR products were purified via the UltraClean PCR clean-up kit (Mo Bio, Carlsbad, CA, USA) following the manufacturer’s instructions, and sequenced by the Southern Pathology Sequencing Facility at Flinders Medical Centre. The neighbor joining tree was constructed using the molecular evolutionary genetic analysis package version 5.0 based on 1000 bootstrap values (Tamura et al., 2011). The presence of the *puf*M gene was identified using published *puf*M primers (Achenbach et al., 2001). The presence of *nif*H gene was also analyzed using a gene specific primer (Cantera et al., 2004). *In silico* analysis was done by using the blast programs to search the GenBank and NCBI databases (http://www.ncbi.nlm.nih.gov).

### Microbial Fuel Cell (MFC) construction

Single chamber and dual chamber cubic MFC reactors were used to evaluate power generation from strain RP2. Single chamber bottle MFCs were made from laboratory Schott duran bottles with a capacity of 300 ml as suggested by Logan et al. (2007). The liquid volume of the chamber was 280 ml. Dual chamber cubic MFCs were constructed using two lexan glasses separated by a cation exchange membrane (Membrane International Inc., USA). The liquid volume of a dual chamber cubic MFC was 150 ml. Anodes were carbon paper or graphite fiber brushes of 5 cm in diameter and 7 cm in length. The graphite brushes were treated as previously described (Feng et al., 2010). Plain carbon paper was used as the anode material in dual chamber cubic MFC systems. Single chamber air cathodes contained 0.5 mg cm^-2^ of platinum catalyst on the liquid facing side with diffusional layers of PTFE applied to the air facing side (Cheng et al., 2006). All the reactors were sterilized before use.

### MFC Operational Conditions

Strain RP2 was used for fuel cell experiments with acetate (1 g/L) as the electron donor in 50 mM PBS buffer. A higher concentration (200 mM) of buffer was also used in fuel cell experiments to examine the effect on power generation. A stationary phase culture was used in the anode chamber and the cells were operated under illuminated conditions. The anodic chamber was flushed with nitrogen gas and filled with anaerobic growth medium. The anolyte was agitated using a magnetic stirrer operating at 100 rpm. In the cubic MFC, the cathode chamber was provided with air through a 0.45 μ pore sized membrane filter. Solutions were replaced when the voltage dropped <10 mV. Open circuit MFC studies were also carried out and then switched to closed circuit with a selected external load (R-1000 Ω unless stated otherwise). DRH compounds were also used as sole source of energy in degradation experiments using the strain RP2 at a concentration of 800 mg l^-1^ in MERS studies. All the reactors were maintained in photoheterotrophic and chemoheterotrophic culture conditions at room temperature in triplicates.

### Electrochemical Analysis

The electrochemical activity of strain RP2 was examined using cyclic voltammetry with a conventional three electrode electrochemical cell with a 25 ml capacity as described earlier (Kim et al., 1999). Bacterial cells grown (aerobic and anaerobic growth environments) in Fe(III) oxide liquid cultures were harvested and used for testing electrochemical activities. Cyclic voltammograms of the bacterial suspension were obtained using a potentiostat (Electrochemical analyser, BAS 100B, USA) connected to a personal computer with BAS software. A glassy carbon working electrode (3 mm, diameter, MF-2012, BAS) and silver/silver chloride reference electrode (MW-4130, BAS) and platinum counter electrode (MW-4130, BAS) were used in a conventional three electrode system. The working electrodes were polished with alumina slurry on cotton wool before each measurement. The electrochemical cells were purged with nitrogen gas for 15 minutes before each measurement. Different scan rates were used from 5 to 100 mV/sec with a potential range from -800 to 800 mV.

### Analytical Methods and Calculations

Fe(III) reduction was monitored by measuring Fe(II) production by the ferrozine method (Lovley and Phillips, 1988b). The DRH was extracted in acetone-methylene chloride (1:1) mixture, dewatered and concentrated by evaporator, and then concentrations were measured by GC-FID using a HP-5 capillary column (15 m length, 0.32 mm thickness, 0.1 μm internal diameter) following the USEPA protocol (USEPA, 1996). The resulting chromatograms were analyzed using Agilent software (GC-FID Agilent model 6890) to identify the hydrocarbon degradation products. Chemical oxygen demand was measured by COD analyser (Chemetrics, K-7365). Fuel cell power output was monitored using a DMM (Keithly Model 2701, USA) linked to a multi-channel scanner (Module 7700, Keithly Instruments, USA). Data were recorded digitally on an Intel computer via IEEE 488 input system and Keithly cable. To measure the current under closed circuit conditions, the external load was connected (R-1000Ω unless stated otherwise). Current was calculated by using *I* = *V/R*, and power was calculated via *P* = *VI*. Power density and current density were normalized to the projected surface area of a cathode. Polarization curves were plotted using various external loads with a range of 10 Ω to open circuit. Coulombic efficiency (CE) was calculated at the end of the cycle from COD removal as previously described by an Logan (2008).

### Nucleotide Sequence Numbers

The 16S rRNA, *puf*M, *nif*H gene sequences have been deposited in the GenBank database under the accession numbers of KJ460004, J1289478, J223658.

## Results

### Strain Identification

In our laboratory experiments, a strain of *R. palustris* strain RP2 emerged as a dominant species was isolated from the anodic biofilm PH fed MERS. *R. palustris* strain RP2 is a cosmopolitan phototrophic bacterium, contains double membrane bilayers, lamellar thylakoid membrane system, produce chains of magnetosomes (Venkidusamy et al., 2015), and grow as long bacillus-shaped (0.5 to 1 μm wide and 2.0 to 6 μm long cells) with asymmetric cell division. Cell division occurred by budding, with dumbbell shaped daughter cells, forming rosette-like structures. Thin section transmission electron micrographs revealed the presence of a lamellar thylakoid membrane system presumably containing the photosynthetic apparatus in anoxic phototrophically grown cells (Supplementary Figure S1A) whereas cells grown in the dark lacked intra cytoplasmic (ICM) membranes (Supplementary Figure S1B).

### Photosynthetic Pigments

Photosynthetically grown anoxic liquid cells were dark red (Supplementary Figure S2A) in color whereas chemosynthetically grown anoxic cells were colorless (Supplementary Figure S2B). Cultures grown under aerobic conditions were faint pink to colorless. Thin section transmission electron micrographs revealed the presence of a lamellar thylakoid membrane system presumably containing the photosynthetic apparatus in anoxic phototrophically grown cells. Absorption maxima of the homogenized photosynthetic cells showed three bacteriochlorophyll peaks at 379, 473, and 503 nm, and peaks at 545, 594, 807, and 875 nm indicated the presence of carotenoid pigments of the spillriloxanthin series. In contrast, no peaks were observed in the chemosynthetically grown cells. The presence of the photosynthetic gene (*puf*M gene) in the strain RP2 was shown using PCR with primers specific for this gene, resulting in a 230 bp product.

### Physiological and Metabolic Characters

**FIGURE 1:**
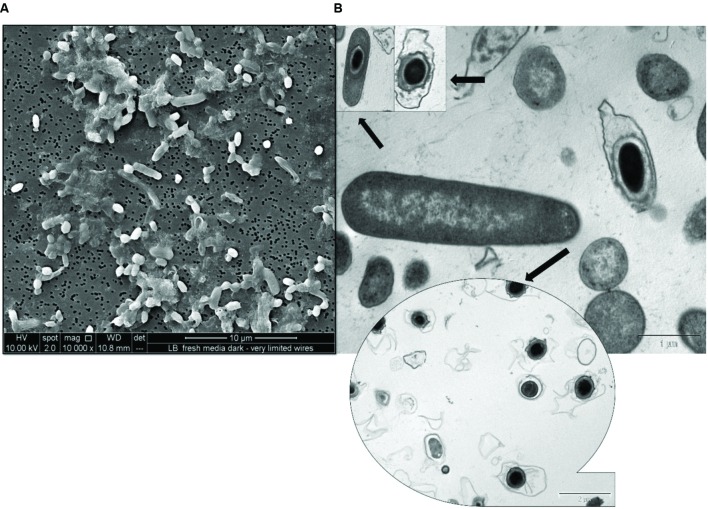
**Micrographs of spore formation in chemosynthetically grown cells of *R. palustris* strain RP2. (A)** SEM micrograph. **(B)** TEM micrograph.

The cells of strain RP2 have exceptionally flexible growth based on environmental signals such as photoorganotrophic, photolithotrophic, dark fermentative, and aerobic heterotrophic mechanisms. Optimum bacterial growth was observed at 25 to 30°C at a neutral pH, whilst no growth was detected above 40°C. Experiments to determine the growth factor requirements for the strain RP2 clearly shows the need for *p*-aminobenzoate, pyridoxine HCl, and folic acid (Supplementary Table S1). Salient properties of the strain RP2 were direct electrode respiration, dissimilatory metal oxide reduction, anaerobic nitrate reduction, carbon dioxide fixation, free living diazotrophy, and the ability to degrade n-alkane components of PH under anoxic environments. Strain RP2 showed growth in minimal medium in the absence of both nitrogen and carbon sources under anoxic, photosynthetic conditions. Nitrogen fixation trait was confirmed by the detection of a *nif*H gene required for nitrogen fixation in the strain. The strain RP2 differed from a previously reported exoelectrogenic strain of *R. palustris* DX1 (Xing et al., 2008) with respect to its ability of photoassimilating a range of substrates including gluconate, aspartate, glycerol, and amino-ethanol. However, the strain RP2 was unable to utilize some compounds such as sebacic acid, threonine, L-ornithine, and L-proline. The ability to utilize sodium benzoate distinguishes the strain from other species of purple sulfur bacteria. The strain RP2 can assimilate acetate photosynthetically with nitrate, sulfate, and iron as terminal electron acceptors (Supplementary Figure S3). We cultured RP2 cells under two different environmental conditions: anoxic photoheterotrophic and anoxic chemoheterotrophic with crystalline Fe(III) oxide as a terminal electron acceptor to investigate the dissimilatory metal oxide reduction trait. Fe(III) oxide reduction was monitored by color change and hydroxylamine Fe(II)extraction assay. Fe(III) oxide reduction of 69.5% ± 0.41% was observed only in anoxic photoheterotrophic environments (Venkidusamy et al., 2015) whereas chemosynthetic grown cells showed no reduction. During this process, peculiar extracellular electrically conductive nanofilamentous structures were observed in the phototrophic growth conditions as stated previously (Venkidusamy et al., 2015). When colonies are incubated for longer incubations (for a period of 4-5 weeks) under anoxic chemosynthetic conditions, they develop a complex morphology such as spore formation with a well-defined outer layer (**Figure 1**).

### Phylogeny

**FIGURE 2:**
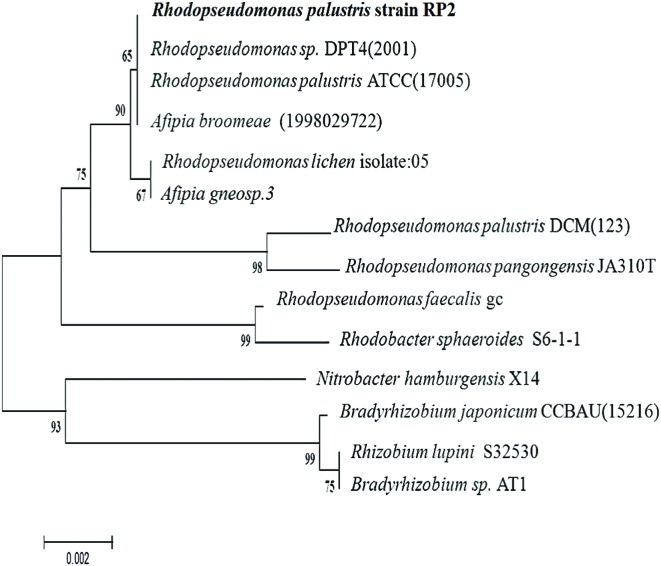
**Phylogenetic tree based on 16S rRNA sequences showing the positions of the isolate, *R. palustris* strain RP2 and representatives of other closely related species.** The tree was constructed form 1,420 aligned bases; Scale bar represents 0.002 substitution per nucleotide.

ClustalW alignment was used to align 1420 bp of the 16S ribosomal RNA gene sequence of strain RP2 with the same region of several related non-sulfur alphaproteobacteria groups. Using this multiple alignment, the neighborhood phylogenetic tree was constructed (**Figure 2**). Phylogenetic analysis of 16S rRNA gene sequences of strain RP2 demonstrated that the strain was closely linked to the genera of *Rhodopseudomonas* with 98% identity to sequences from *R. palustris* ATCC (17005) and *Rhodopseudomonas* sp. DPT4 (2001).

### Current Generation by *R. palustris* Strain RP2 in Acetate MFCs

**FIGURE 3:**
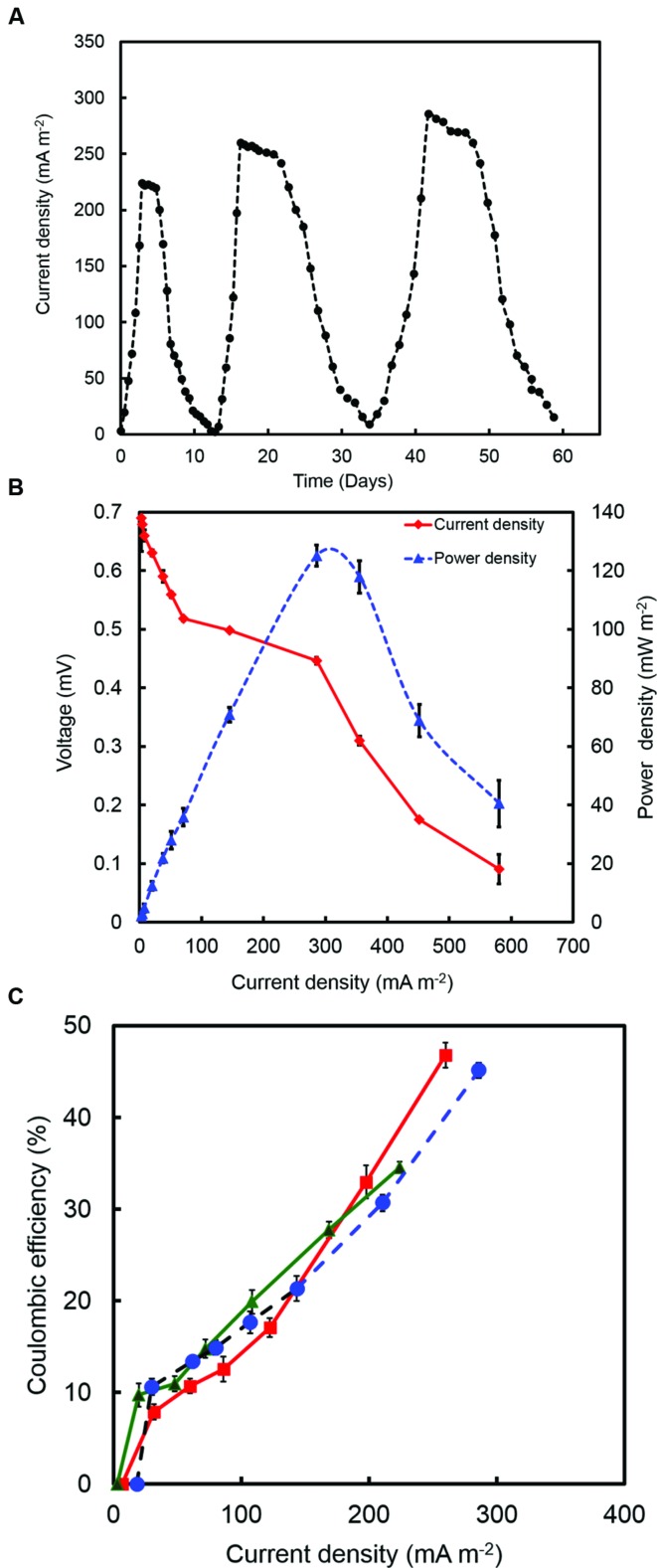
**(A)** A few representative initial cycles of current density (average from triplicates) generated by strain RP2 fed with acetate. **(B)** Power density and voltage generation as a function of current density from strain RP2 inoculated acetate-fed bottle MFCs. **(C)** Coulombic efficiency (CE) of a few representative batches of acetate-fed bottle MFCs.

Current was generated in all the MFCs inoculated with strain RP2 using acetate as an energy source. After three days, voltage started to follow a constant pattern and then stabilized. The fuel cell electrodes were discharged through a 1000 Ω external resistance once it reached the plateau voltage generation stage. The voltage fell quickly from 690 ± 10 mV to 446 ± 5 mV after a 1000 Ω fixed resistor was connected. The maximum output range of voltage and current density were 446 ± 7 mV, 305 ± 10 mA/m^2^ (*R* = 1000 Ω) after four cycles of operation. Few representative initial cycles (average current density from triplicates) of current density are shown in (**Figure 3A**). After six refilling batches with a fresh substrate, the maximum current output of each batch became stable (300 ± 7 mA/m^2^). The maximum open circuit voltage was 700 ± 7 mV and attainable power density was 131.65 ± 10 mW/m^2^ (*R* = 1000 Ω) (**Figure 3B**). The CEs showed the increased trend with the increased current densities as shown in the **Figure 3C**. The maximum CE was 46.7% which corresponded to the maximum current density of 259.90 mA/m^2^. The higher buffer (200 mM) concentration were also examined at the same resistance which produced a maximum power density of 300 ± 20 mW/m^2^.

**FIGURE 4:**
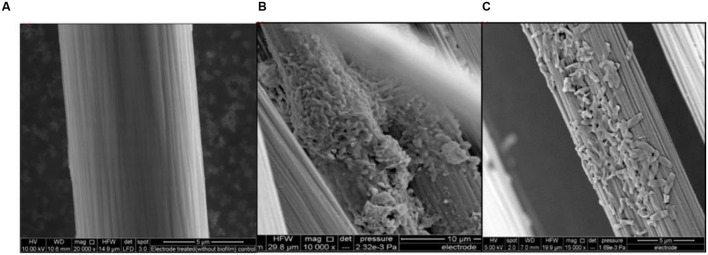
**Anode micrographs of *R. palustris* strain RP2 inoculated acetate-fed MFCs. (A)** Treated control anode, **(B)** Strain RP2- biofilm on graphite surface and **(C)** Cells with high magnification.

Experiments were also conducted using dual chamber cubic MFCs (CMFC) containing carbon flat paper anodes at a fixed resistance of 1000 Ω. The voltage profile of the cubic MFC revealed that the voltage produced was less than that for the air cathode MFC. A long lag time was observed with the carbon paper anode whereas a treated brush anode reduced the strain acclimation period and resulted in an increased power generation. The maximum open circuit potential and power output of CMFC were 600 ± 7 mV and 70 mW/m^2^, respectively. Constant power and voltage output indicated the formation of a stable biofilm around the electrode surface. Scanning electron micrographs revealed that the bacterial population from the graphite brush anode was homogenous in shape (**Figure 4**).

### Electrochemical Activity of *R. palustris* Strain RP2

**FIGURE 5:**
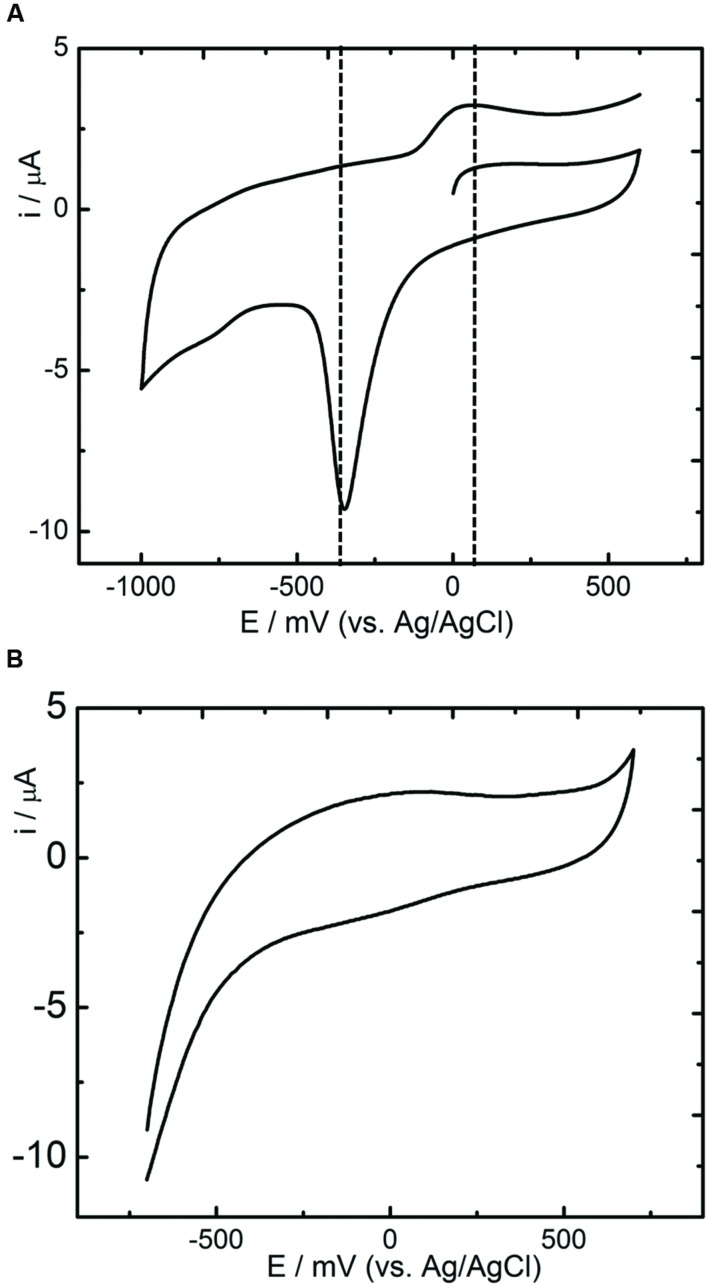
**Cyclic Voltammetry studies of *R. palustris* strain RP2. (A)** Anoxic grown cells; **(B)** Aerobically grown cells; Scan rate: 5 mV s^-1^.

Cell suspensions were prepared from the bacterial cells grown under anaerobic and aerobic environments to determine the electrochemical activities of the strain RP2 using cyclic voltammetry (CV) studies. Oxidation and reduction peaks of CV were observed in anaerobic grown bacterial cells (**Figure 5A**) whereas aerobically grown cells lacked the electrochemical activity (**Figure 5B**). Reduction peaks ranging from -394 mV to -399 mV and oxidation peaks ranging from -200 mV to +100 mV were observed at the electrode interface from washed cells suspensions. The calculated mid-point potential was about -278 mV. This asymmetric CV peak shows that the redox reaction is a quasi-reversible reaction. The amplitude of the peaks increased according to the growth stage of the culture in batch mode. The highest peaks were present during the exponential growth stage which indicated that the development of biofilm was the main factor for the electron transfer. One redox couple was observed from the CV peak and number of electrons transferred was calculated based on the Nernst equation (Logan, 2008). The CV peaks were not observed from the suspension of aerobically grown cells or autoclaved controls.

### Hydrocarbonosclastic Potential of *R. palustris* Strain RP2 in Anoxic Environments

#### Degradation of Hydrocarbons in Anoxic, Photosynthetic Incubations

**FIGURE 6:**
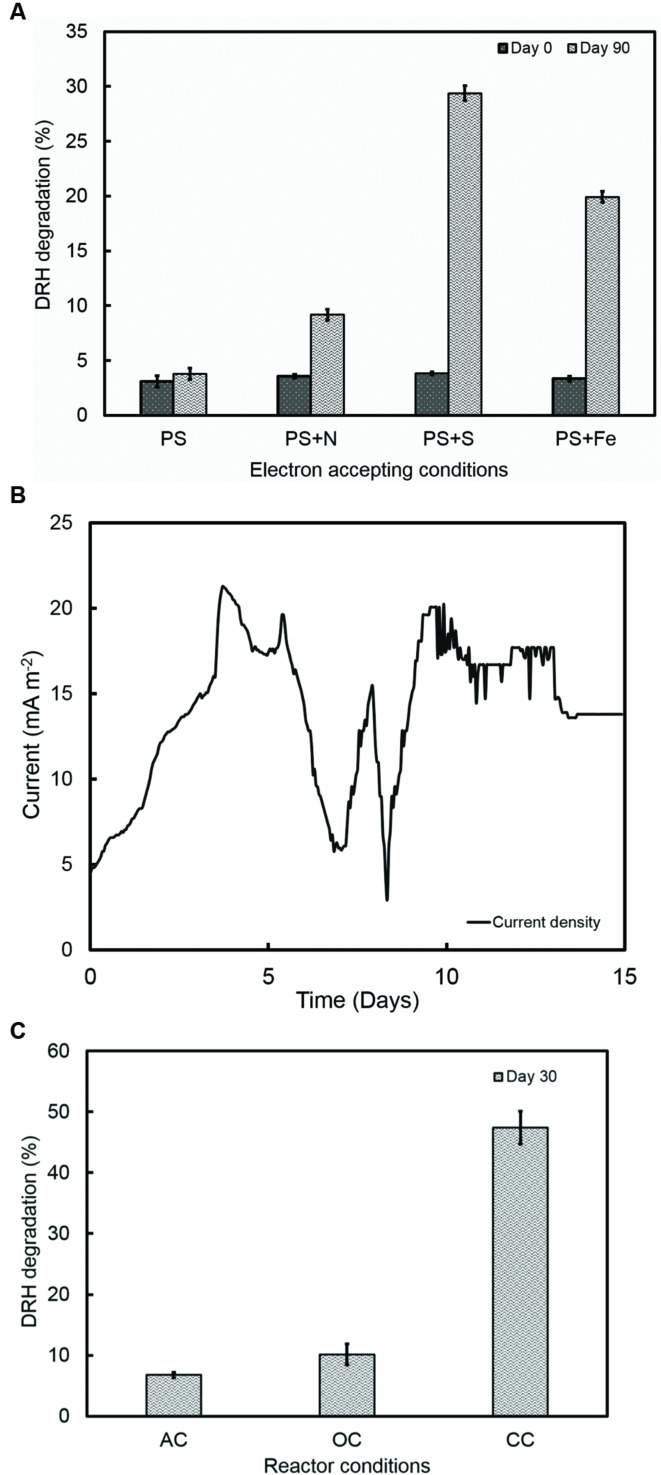
**(A)** Photoorganotrophic hydrocarbon degradation under different electron accepting conditions (PS-photosynthetic; PS+N- Photosynthetic + Nitrate; PS+S; Photosynthetic + Sulfate; PS+Fe- Photosynthetic + Iron). **(B)** A representative current density cycle of diesel fed microbial fuel cell system using strain RP2. **(C)** Hydrocarbon degradation under different reactor operating conditions (AC, Abiotic controls; OC, Open circuit controls; CC- Closed Circuit).

To study the hydrocarbon degradation potential of the strain RP2, experiments were performed under four different environments: oxic, anoxic, photosynthetic, and chemosynthetic. Cultures were inoculated into Wolfe’s media with DRHs as a sole carbon source with different electron accepting conditions. These incubation experiments indicate that photosynthetic anaerobic degradation of hydrocarbons occurred, however, no increase in biomass or hydrocarbon degradation was observed in chemosynthetically grown anaerobic or aerobic samples. The rate and extent of biodegradation was interpreted from GC chromatograms of the residual hydrocarbons. **Figure 6A** shows the possible anoxic photoheterotrophic degradation of DRH compounds under different electron accepting conditions [Photosynthetic only (PS); Photosynthetic + Nitrate (PS+N); Photosynthetic + Sulfate (PS+S); Photosynthetic + Iron (PS+Fe)]. For a substrate concentration of 4000 mg/l, cells showed ~10 to 30% of DRH removal by the end of the experiment. A higher percentage of DRH removal (30.6 ± 0.68%, 90th day) was noticed in the triplicates of sulfate containing anoxic, photoheterotrophic samples. Abiotic loss of DRH was measured under each condition was less than 5%.

#### Degradation of Hydrocarbons in MERS Environments

The ability of the strain RP2 to produce electricity using DRH as a sole carbon source was also examined using an initial concentration of 800 mg l^-1^ of DRHs for three complete cycles. A lag time of 90 h was observed in DRH fed MERS before a constant current was established. A maximum current and power density generated at this concentration were 21 ± 3 mA/m^2^ (**Figure 6B**), 720 ± 7 μW/m^2^. An average of 47.4 ± 2.7% decrease in DRH was observed in closed circuit MERS inoculated with the strain RP2 by the end of experiment (30days). In the case of the abiotic (AC) and open circuit (OC) controls, DRH removal rates were 6.7 ± 0.43% and 10.1 ± 1.7% by the end of experiment. The current density and degradation profiles suggest that MERS could utilize the DRH as a sole source of energy generation which indicated the acclimation of strain RP2 biofilm (**Figure 6C**). However, no increase in biomass or hydrocarbon degradation or current generation was observed in chemosynthetically incubated MERS samples.

## Discussion

*Rhodopseudomonas* species are associated with a variety of environments such as limnetic zones (Eckersley and Dow, 1980), marine environments (Larimer et al., 2004), sewage sludges (Hiraishi and Ueda, 1995), euxinic lagoons (Whittenbury and McLee, 1967), and poorly drained soils. The strain RP2 is a facultatively anaerobic, Gram negative purple non-sulfur photosynthetic rod shaped bacterium, containing lamellar ICMs as previously described (Mehrabi et al., 2001). It is a magnetotactic bacterium (Vainshtein et al., 1997), capable of nitrogen (Cantera et al., 2004), and carbon dioxide fixation (Larimer et al., 2004) as reported earlier. The detection of the *nif* H and *puf*M genes confirmed the presence of diazotrophic and photosynthetic traits as stated previously (Achenbach et al., 2001; Oda et al., 2005). With respect to this, it shares general characteristics with the *Rhodopseudomonas* genus of Rhodopspirillaceae. However, a few distinctive features make this strain different from the existing members of the family, these include photoheterotrophic anoxic Fe(III) oxide reduction, electrically conductive filaments (Venkidusamy et al., 2015), spore formation, (**Figure 1**) growth factor requirements and the ability to degrade *n*-alkane components of DRHs in anoxic and MERS environments.

The normal growth mode of photosynthetic purple non-sulfur bacteria is photoheterotrophic; however, strain RP2 is able to switch to other metabolic modes such as photoorganoheterotrophic, phtotoorganoautotrophic, chemorg- anoheterotrophic, photolithoautotrophic, and chemoautotrophic growth. The strain RP2 also showed photolithotrophic growth with thiosulphate as an electron donor whereas pure cultures of *R. blastica* cannot utilize thiosulphate as an inorganic electron donor (Eckersley and Dow, 1980). Aerobic growth of the strain RP2 was also possible under both illuminated and non-illuminated conditions; however, strains of *Rhodopsedumonas* such as *R. faecalis* (Zhang et al., 2002), and *R. palustris*-17 (Dönmez et al., 1999) are unable to grow in oxygen rich, illuminated environments. The strain RP2 has a quite different pattern of carbon source utilization compared to other members of the genus *Rhodopseudomonas* (Supplementary Table S1). For example, strain RP2 was able to use gluconate and aspartate whereas *R. palustris* DX1 and *R. palustris* TIE-1 were unable to utilize those compounds (Xing et al., 2008). Under anaerobic conditions, the strain RP2 can utilize sodium benzoate as a carbon and energy source whereas the strain *Rhodopseudomons* DCP-3 can oxidize benzoate only under aerobic conditions (Krooneman et al., 1999). The same pattern of glucose utilization under photoheterotrophic conditions has been reported (Imhoff and Bias-lmhoff, 1995), however few strains of *Rhodopseudomonas* are unable to grow when the medium is enriched with glucose as a sole substrate (Dönmez et al., 1999). Different patterns of vitamin requirements including p-amino benzoic acid, folic acid and pyridoxine HCl highlighted differences in the requirements of photosynthetic non-sulfur bacteria (Dönmez et al., 1999). The most interesting feature of the strain is photoheterotrophic ferric oxide reduction which required both light and an organic carbon source. This also provided evidence of a dissimilatory metal oxide reduction pathway (DMRB) similar to that seen in other electrochemically active bacterial strains (Lovley et al., 1993; Bretschger et al., 2007). On the other hand, photoferrotrophic growth of *R. palustris* strain TIE-1 has been demonstrated in the studies of anaerobic oxidation of Fe(II) (Bose et al., 2014; Byrne et al., 2015). Considering these features, this strain RP2 appears to be a novel member of the purple non-sulfur photosynthetic bacterial genus of *Rhodopseudomonas.*

The strain RP2 can transfer the electrons to extracellular, insoluble electron acceptors as reported earlier (Xing et al., 2008; Venkidusamy et al., 2015). Recent investigations have revealed the potential of using such phototrophic biofilms in a mediator free MFC systems (Park et al., 2014; Li et al., 2015). For instance, Xing et al. (2008), documented the highest power density of 2.72 ± 0.06 W/m^2^ by *R. palustris* DX1 using dual chamber MFCs which is higher than that produced by mixed biofilms. *R. palustris* micro MFCs fed with renewable substrates such as blue green algae produced more power output of 10.4 mW/m^3^ than other chemical substrates used (Inglesby et al., 2012). The present study showed a maximum power density of 131.65 ± 10 mW/m^2^ (acetate fed MFC, 50 mM buffer concentration) with an electron recovery of more than 40% as current using the strain RP2. However, the maximum power densities by such pure exoelectrogens are considerably influenced by a number of reactor parameters, operating conditions such as electrodes and its distance, electrode surface area to the volume ratio, pH, dissolved oxygen, resistance, electrolytes, etc. as reported earlier (Min et al., 2005; Logan, 2008). Biofilms and supernatant of the strain RP2 was used to determine the electrochemical activity through the release of redox proteins using CV analysis (**Figures 5A,B**). These results suggest that the presence of redox compounds in strain RP2 may be involved in extracellular electron transfer. The CV of *R. palustris* strain RP2 biofilm with Fe(III) oxide revealed the distinct redox peaks at -398 mV, +150 mV. The mid-potential of the CV peaks obtained matched the outer membrane cytochromes (OmcA) that have redox potential around -240 to -320 mV as reported in *Shewanella oniendensis* MR (Kim et al., 2002), *Geobacter*, *Desulfuromonas acetoxidans* (Lojou and Bianco, 2004). Further, CV curves from a bacterial cell suspension showed a smaller cell potential value than the theoretical electrode potential which was responsible for poor performance and operating conditions of MFCs (Kim et al., 1999). The results also suggest that oxygenated liquid cultures prevent the synthesis of the outer membrane cytochromes which plays important role in extracellular electron transfer to insoluble metal oxides.

### Metabolic Specialization and Biotechnological Potential of *R. palustris* Strain RP2

The genus *Rhodopseudomonas* has been studied as a versatile bioremediation candidate because of its exceptional growth flexibility involving the metabolism of diverse compounds. These include utilization of N-aromatic rings (Dutton and Evans, 1969), heterocyclic compounds, chlorinated compounds (McGrath and Harfoot, 1997), heavy metals and other groups of xenobiotic pollutants, for instance, phenolic compounds (Mehrabi et al., 2001), and other dihydroxylated aromatic aldehydes (Harwood and Gibson, 1986). However, the degradation of aliphatic hydrocarbons compounds by the genus *Rhodopseudomonas* is previously unknown. It was demonstrated here for the first time that the strain RP2 is capable of mineralizing DRHs in anaerobic environments.

In order to assess the hydrocarbon degradation potential of the strain RP2, GC scan was performed using the photoheterotrophicaly grown samples grown under different electron accepting conditions. We showed evidence for the existence of anaerobic hydrocarbon degrading capability in the strain RP2 when grown under illuminated conditions in the presence of thiosulfate. To obtain deeper insights into hydrocarbon degradation mechanism by the strain RP2, further research is required in terms of catabolic genes encoding alkane degrading enzymes such as Cytochrome 450, *alk* genes including *alk*B, *alk*M, *alk*A etc., The presence of such specific catabolic genes was investigated using the PCR mediated amplification method with various oligonucleotide primers (Supplementary Table S3). This strain, however, showed negative and non-specific results for CYP153A, *alk*B and its related, *alk*M and *alk*A genes. Efforts are being made using *R. palustris* strain RP2 genome sequence analysis to improve our understanding of anoxic photoorganotrophic hydrocarbon degradation mechanism in strain RP2. Metabolic prediction analysis using KEGG (Kyoto encyclopedia of Genes and Genome) reveals the presence of key enzymes involved in biodegradation of hydrocarbons. Supplementary Table S2 shows the comparison of metabolic genes present in the genome of *R. palustris* strain RP2 with other genomes obtained from database. Results of this study shows that the strain RP2 possesses alkane sulfonate monooxygenase and catechol 1,2-dioxygenase for hydrocarbon degradation as recently mentioned in *Pseudomonas aeruginosa* strain N0002 (Roy et al., 2013).

Previously, the remediation of hydrocarbons in MERS systems required mixed biofilms or enriched biofilms (Morris et al., 2009; Venkidusamy et al., 2016). The present study also demonstrated the potential of generating current in the presence of hydrocarbon compounds using the strain RP2 in MERS for the first time. However, the maximum current and power densities of hydrocarbons fed MERS were much lesser than the mixed culture MERS studies using freshly inoculated anodes (70.57 mA/m^2^ at a concentration of 800 mg L^-1^) (Venkidusamy et al., 2016), suggesting that the lower rate of hydrocarbons assimilation (47.4 ± 2.7% by 30th day) limited the power generation. Whereas mixed culture MERS system showed nearly 84% of DRH removal at this concentration by the end of batch experiment (Venkidusamy et al., 2016). This perhaps indicates the presence of microbial interactions and its synergistic effects between different species in the mixed culture MERS systems. Interestingly, the strain RP2 also showed the physiological induction of electrically conductive nanofilaments in energetically engineered environments as recently reported (Venkidusamy et al., 2015). It will be interesting to examine whether the induction of these nanofilaments are involved in enhanced bioremediation of hydrocarbon contaminants in energetically engineered environments.

## Conclusion

The members of *Rhodopseudomonas* species appear to have a cosmopolitan distribution as their presence has been detected in marine environments. The present study demonstrated for the first time, the hydrocarbon bioremediation potential of *R. palustris* strain RP2 in anaerobic and MERS environments. The findings of this study not only expand our knowledge of the range of bacteria known to degrade hydrocarbon contaminants but also provide further research opportunities in the field of sustainable remediation (MERS), molecular biology of hydrocarbon bio-degradative genes in EABs and their interactions in oil contaminated sites. Thus, this study will undoubtedly contribute to the biotechnological applications involved in advanced bioremediation techniques (MERS) and bioremedial process of hydrocarbons at photic, marine environments.

## Author Contributions

KV and MM proposed the study. KV conducted the experiments under the supervision of MM. KV prepared the draft with contributions from MM.

## Conflict of Interest Statement

The authors declare that the research was conducted in the absence of any commercial or financial relationships that could be construed as a potential conflict of interest.

## References

[B1] Achenbach L. A. Carey J. Madigan M. T. (2001). Photosynthetic and phylogenetic primers for detection of anoxygenic phototrophs in natural environments. *App. Environ. Microbiol.* 67 2922–2926. 10.1128/AEM.67.7.2922-2926.2001 PMC92962 11425703

[B2] Bose A. Gardel E. J. Vidoudez C. Parra E. Girguis P. R. (2014). Electron uptake by iron-oxidizing phototrophic bacteria. *Nature commun.* 5 3391 10.1038/ncomms4391 24569675

[B3] Bretschger O. Obraztsova A. Sturm C. A. Chang I. S. Gorby Y. A. Reed S. B. (2007). Current production and metal oxide reduction by *Shewanella oneidensis* MR-1 wild type and mutants. *App. Environ. Microbiol.* 73 7003–7012. 10.1128/AEM.01087-07 PMC2074945 17644630

[B4] Byrne J. M. Klueglein N. Pearce C. Rosso K. M. Appel E. Kappler A. (2015). Redox cycling of Fe (II) and Fe (III) in magnetite by Fe-metabolizing bacteria. *Science* 347 1473–1476. 10.1126/science.aaa4834 25814583

[B5] Cantera J. J. L. Kawasaki H. Seki T. (2004). The nitrogen-fixing gene (nifH) of *Rhodopseudomonas palustris*: a case of lateral gene transfer? *Microbiol.* 150 2237–2246. 10.1099/mic.0.26940-0 15256566

[B6] Chaudhuri S. K. Lovley D. R. (2003). Electricity generation by direct oxidation of glucose in mediatorless microbial fuel cells. *Nat. Biotechnol.* 21 1229–1232. 10.1038/nbt867 12960964

[B7] Cheng S. Liu H. Logan B. E. (2006). Increased performance of single-chamber microbial fuel cells using an improved cathode structure. *Electrochem. Commun.* 8 489–494. 10.1016/j.elecom.2006.01.010

[B8] Dönmez G. Ç. Öztürk A. Çakmakçi L. (1999). Properties of the *Rhodopseudomonas palustris* strains isolated from an alkaline lake in Turkey. *Turkish J. Biol.* 23 457–464.

[B9] Dutton P. Evans W. (1969). The metabolism of aromatic compounds by *Rhodopseudomonas palustris*. *Biochem. J.* 113 525–536. 5807211 10.1042/bj1130525 PMC1184695

[B10] Eckersley K. Dow C. S. (1980). *Rhodopseudomonas* blastica sp. nov.: a member of the *Rhodospirillaceae*. *J. Gen. Microbiol.* 119 465–473. 10.1099/00221287-119-2-465

[B11] Feng Y. Yang Q. Wang X. Logan B. E. (2010). Treatment of carbon fiber brush anodes for improving power generation in air–cathode microbial fuel cells. *J. Power Sources* 195 1841–1844. 10.1016/j.jpowsour.2009.10.030

[B12] Harwood C. S. Gibson J. (1986). Uptake of benzoate by *Rhodopseudomonas palustris* grown anaerobically in light. *J. bacteriol.* 165 504–509. 3944059 10.1128/jb.165.2.504-509.1986 PMC214447

[B13] Hiraishi A. Ueda Y. (1995). Isolation and characterization of *Rhodovulum strictum* sp. nov. and some other purple nonsulfur bacteria from colored blooms in tidal and seawater pools. *Int. J. Syst. Bacteriol.* 45 319–326. 7537066 10.1099/00207713-45-2-319

[B14] Holmes D. E. Chaudhuri S. K. Nevin K. P. Mehta T. Methé B. A. Liu A. (2006). Microarray and genetic analysis of electron transfer to electrodes in *Geobacter sulfurreducens*. *Environ. Microbiol.* 8 1805–1815. 10.1111/j.1462-2920.2006.01065.x 16958761

[B15] Huang L. Cheng S. Chen G. (2011). Bioelectrochemical systems for efficient recalcitrant wastes treatment. *J. Chem. Technol. Biotechnol.* 86 481–491. 10.1002/jctb.2551

[B16] Hungate R. (1950). The anaerobic mesophilic cellulolytic bacteria. *Bacteriol. Rev.* 14 1–49. 15420122 10.1128/br.14.1.1-49.1950 PMC440953

[B17] Imhoff J. F. Bias-lmhoff U. (1995). “Lipids, quinones and fatty acids of anoxygenic phototrophic bacteria,” in *Anoxygenic Photosynthetic Bacteria* eds Blankenship R. E. Madigan M. T. Bauer C. E. (Dordrecht: Springer) 179–205.

[B18] Inglesby A. E. Beatty D. A. Fisher A. C. (2012). *Rhodopseudomonas palustris* purple bacteria fed *Arthrospira maxima* cyanobacteria: demonstration of application in microbial fuel cells. *RSC. Adv.* 2 4829–4838. 10.1039/c2ra20264f

[B19] Kim B.-H. Kim H.-J. Hyun M.-S. Park D.-H. (1999). Direct electrode reaction of Fe (III)-reducing bacterium, *Shewanella putrefaciens*. *J. Microbiol. Biotechnol.* 9 127–131. 10.1371/journal.pone.0147899

[B20] Kim H. J. Park H. S. Hyun M. S. Chang I. S. Kim M. Kim B. H. (2002). A mediator-less microbial fuel cell using a metal reducing bacterium, *Shewanella putrefaciens*. *Enzyme Microb. Technol.* 30 145–152. 10.1016/S0141-0229(01)00478-1

[B21] Kranz R. G. Haselkorn R. (1986). Anaerobic regulation of nitrogen-fixation genes in *Rhodopseudomonas* capsulata. *Proc. Natl. Acad. Sci. U.S.A.* 83 6805–6809. 10.1073/pnas.83.18.6805 3018747 PMC386598

[B22] Krooneman J. van den Akker S. Gomes T. M. P. Forney L. J. Gottschal J. C. (1999). Degradation of 3-chlorobenzoate under low-oxygen conditions in pure and mixed cultures of the anoxygenic photoheterotroph *Rhodopseudomonas palustris* DCP3 and an aerobic *Alcaligenes* species. *App. Environ. Microbiol.* 65 131–137. 10.1128/aem.65.1.131-137.1999 PMC90993 9872770

[B23] Larimer F. W. Chain P. Hauser L. Lamerdin J. Malfatti S. Do L. (2004). Complete genome sequence of the metabolically versatile photosynthetic bacterium *Rhodopseudomonas palustris*. *Nature Biotechnol.* 22 55–61. 10.1038/nbt923 14704707

[B24] Li X. Liu T. Wang K. Waite T. D. (2015). Light-induced extracellular electron transport by the marine Raphidophyte *Chattonella* marina. *Environ. Sci. Technol.* 49 1392–1399. 10.1021/es503511m 25569116

[B25] Logan B. Cheng S. Watson V. Estadt G. (2007). Graphite fiber brush anodes for increased power production in air-cathode microbial fuel cells. *Environ. Sci. Technol.* 41 3341–3346. 10.1021/es062644y 17539547

[B26] Logan B. E. (2008). *Microbial Fuel Cells.* Hoboken, NJ: John Wiley & Sons.

[B27] Lojou E. Bianco P. (2004). Membrane electrodes for protein and enzyme electrochemistry. *Electroanalysis* 16 1113–1121. 10.1002/elan.200403001

[B28] Lovley D. R. Giovannoni S. J. White D. C. Champine J. E. Phillips E. Gorby Y. A. (1993). *Geobacter metallireducens* gen. nov. sp. nov., a microorganism capable of coupling the complete oxidation of organic compounds to the reduction of iron and other metals. *Archiv. Microbiol.* 159 336–344. 10.1007/BF00290916 8387263

[B29] Lovley D. R. Phillips E. J. (1988a). Manganese inhibition of microbial iron reduction in anaerobic sediments. *Geomicrobiol. J.* 6 145–155. 10.1080/01490458809377834

[B30] Lovley D. R. Phillips E. J. (1988b). Novel mode of microbial energy metabolism: organic carbon oxidation coupled to dissimilatory reduction of iron or manganese. *App. Environ. Microbiol.* 54 1472–1480. 10.1128/aem.54.6.1472-1480.1988 PMC202682 16347658

[B31] McGrath J. E. Harfoot C. G. (1997). Reductive dehalogenation of halocarboxylic acids by the phototrophic genera *Rhodospirillum* and *Rhodopseudomonas*. *Appl. Environ. Microbiol.* 63 3333–3335. 9251226 10.1128/aem.63.8.3333-3335.1997 PMC168637

[B32] Mehrabi S. Ekanemesang U. M. Aikhionbare F. O. Kimbro K. S. Bender J. (2001). Identification and characterization of *Rhodopseudomonas* spp., a purple, non-sulfur bacterium from microbial mats. *Biomol. Eng.* 18 49–56. 10.1016/S1389-0344(01)00086-7 11535416

[B33] Min B. Kim J. Oh S. Regan J. M. Logan B. E. (2005). Electricity generation from swine wastewater using microbial fuel cells. *Water Res.* 39 4961–4968. 10.1016/j.watres.2005.09.039 16293279

[B34] Morris J. M. Jin S. Crimi B. Pruden A. (2009). Microbial fuel cell in enhancing anaerobic biodegradation of diesel. *Chem. Eng. J.* 146 161–167. 10.1016/j.cej.2008.05.028

[B35] Oda Y. Samanta S. K. Rey F. E. Wu L. Liu X. Yan T. (2005). Functional genomic analysis of three nitrogenase isozymes in the photosynthetic bacterium *Rhodopseudomonas palustris*. *J. Bacteriol.* 187 7784–7794. 10.1128/JB.187.22.7784-7794.2005 16267302 PMC1280311

[B36] Oh S. Min B. Logan B. E. (2004). Cathode performance as a factor in electricity generation in microbial fuel cells. *Environ. Sci. Technol.* 38 4900–4904. 10.1021/es049422p 15487802

[B37] Park T.-J. Ding W. Cheng S. Brar M. S. Ma A. P. Y. Tun H. M. (2014). Microbial community in microbial fuel cell (MFC) medium and eﬄuent enriched with purple photosynthetic bacterium (*Rhodopseudomonas* sp.). *AMB Exp.* 4 22 10.1186/s13568-014-0022-2 PMC4052673 24949257

[B38] Roy A. S. Baruah R. Gogoi D. Borah M. Singh A. K. Boruah H. P. D. (2013). Draft genome sequence of *Pseudomonas aeruginosa* strain N002, isolated from crude oil-contaminated soil from Geleky, Assam, India. *Genome Announc.* 1 e00104–e00112. 10.1128/genomeA.00104-12 23405324 PMC3569314

[B39] Sambrook J. Fritsch E. F. Maniatis T. (1989). *Molecular Cloning: A Laboratory Manual* 2nd Edn. Plainview, NY: Cold spring harbor laboratory press.

[B40] Tamura K. Peterson D. Peterson N. Stecher G. Nei M. Kumar S. (2011). MEGA5: molecular evolutionary genetics analysis using maximum likelihood, evolutionary distance, and maximum parsimony methods. *Mol. Biol.Evol.* 28 2731–2739. 10.1093/molbev/msr121 21546353 PMC3203626

[B41] USEPA (1996). *SW-846 Method 8015B-3rd Edn, Updates I, II, IIA and III.: Test Methods for Evaluating Solid Wastes, Physical/Chemical Methods, Superintendent of Documents.* Washington, DC: U.S.Governement printing office.

[B42] Vainshtein M. Suzina N. Sorokin V. (1997). A new type of magnet-sensitive inclusions in cells of photosynthetic purple bacteria. *Sys. Appl. Microbiol.* 20 182–186. 10.1016/S0723-2020(97)80064-1

[B43] Venkidusamy K. Megharaj M. Marzorati M. Lockington R. Naidu R. (2016). Enhanced removal of petroleum hydrocarbons using a bioelectrochemical remediation system with pre-cultured anodes. *Sci. Total Environ.* 539 61–69. 10.1016/j.scitotenv.2015.08.098 26360455

[B44] Venkidusamy K. Megharaj M. Schröder U. Karouta F. Mohan S. V. Naidu R. (2015). Electron transport through electrically conductive nanofilaments in *Rhodopseudomonas palustris* strain RP2. *RSC Adv.* 5 100790–100798. 10.1039/C5RA08742B

[B45] Weisburg W. G. Barns S. M. Pelletier D. A. Lane D. J. (1991). 16S ribosomal DNA amplification for phylogenetic study. *J. Bacteriol.* 173 697–703. 1987160 10.1128/jb.173.2.697-703.1991 PMC207061

[B46] Whittenbury R. McLee A. (1967). *Rhodopseudomonas palustris* and Rh. viridis—photosynthetic budding bacteria. *Arch. Mikrobiol.* 59 324–334. 10.1007/BF00406346 5602470

[B47] Wrighton K. C. Agbo P. Warnecke F. Weber K. A. Brodie E. L. DeSantis T. Z. (2008). A novel ecological role of the Firmicutes identified in thermophilic microbial fuel cells. *ISME J.* 2 1146–1156. 10.1038/ismej.2008.48 18769460

[B48] Xing D. Zuo Y. Cheng S. Regan J. M. Logan B. E. (2008). Electricity generation by *Rhodopseudomonas palustris* DX-1. *Environ. Sci. Technol.* 42 4146–4151. 10.1021/es800312v 18589979

[B49] Zhang D. Yang H. Huang Z. Zhang W. Liu S.-J. (2002). *Rhodopseudomonas faecalis* sp. nov., a phototrophic bacterium isolated from an anaerobic reactor that digests chicken faeces. *Inten. J. Sys. Evol. Microbiol.* 52 2055–2060. 10.1099/00207713-52-6-2055 12508868

[B50] Zuo Y. Xing D. Regan J. M. Logan B. E. (2008). Isolation of the exoelectrogenic bacterium *Ochrobactrum anthropi* YZ-1 by using a U-tube microbial fuel cell. *Appl. Environ. Microbiol.* 74 3130–3137. 10.1128/AEM.02732-07 18359834 PMC2394939

