# Supplementary material for: A Novel Electrophototrophic Bacterium Rhodopseudomonas palustris Strain RP2, Exhibits Hydrocarbonoclastic Potential in Anaerobic Environments
Source: Front Microbiol. 2016 Jul 12;7:1071. doi: 10.3389/fmicb.2016.01071 (PMC4940424; doi:10.3389/fmicb.2016.01071)
Supplement: Supplementary file 1 [file Data_Sheet_1.PDF]

Supplementary Material

A novel electrophototrophic bacterium *Rhodopseudomonas palustris* strain RP2, exhibits hydrocarbonoclastic potential in anaerobic environments

Krishnaveni Venkidusamy[a,b*], Mallavarapu Megharaj[a,b,c]

Correspondence: Dr. Krishnaveni Venkidusamy, krishnaveni.venkidusamy@mymail.unisa.edu.au

Supplementary Figures

Fig. S1. **TEM micrographs of photosynthetic apparatus in *R.palustris* strain RP2** (A) containing the photosynthetic apparatus in anoxic phototrophic cells (B) chemosynthetic cells lack ICMs

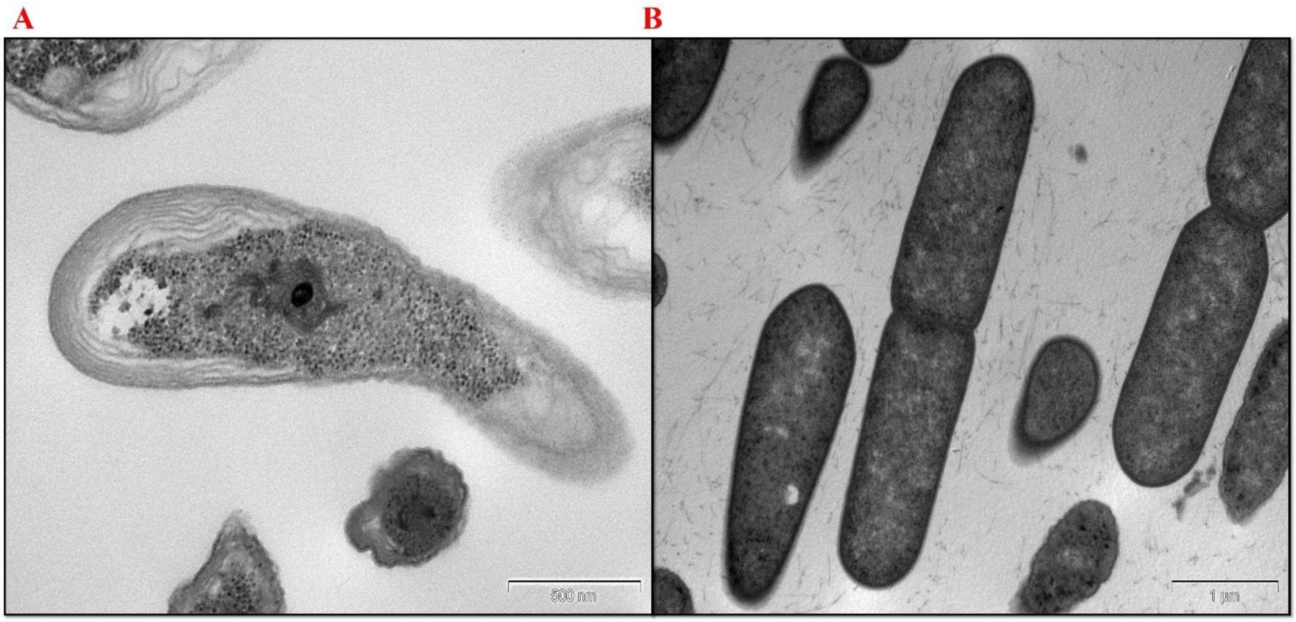

Fig. S2. **Photosynthetic pigments in *R.palustris* strain RP2** (A) Anoxic- photosynthetic cell growth of strain RP2 in LB medium (B) Chemosynthetically grown anoxic cells were colourless; photosynthetically grown anoxic liquid cells were dark red in colour

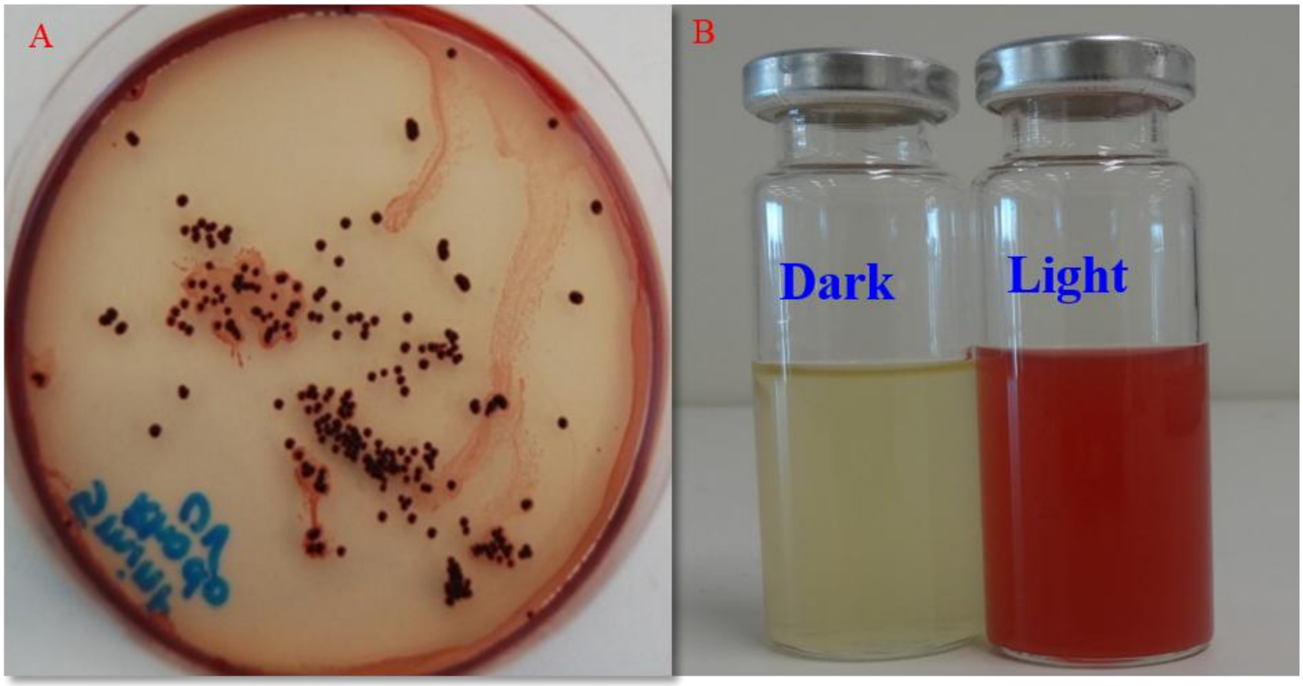

Fig. S3. **Acetate utilisation under different electron accepting conditions by *R.palustris* strain RP2** (NO_3 -Nitrate; SO_4- Sulfate; Fe_3- Iron (III) citrate)

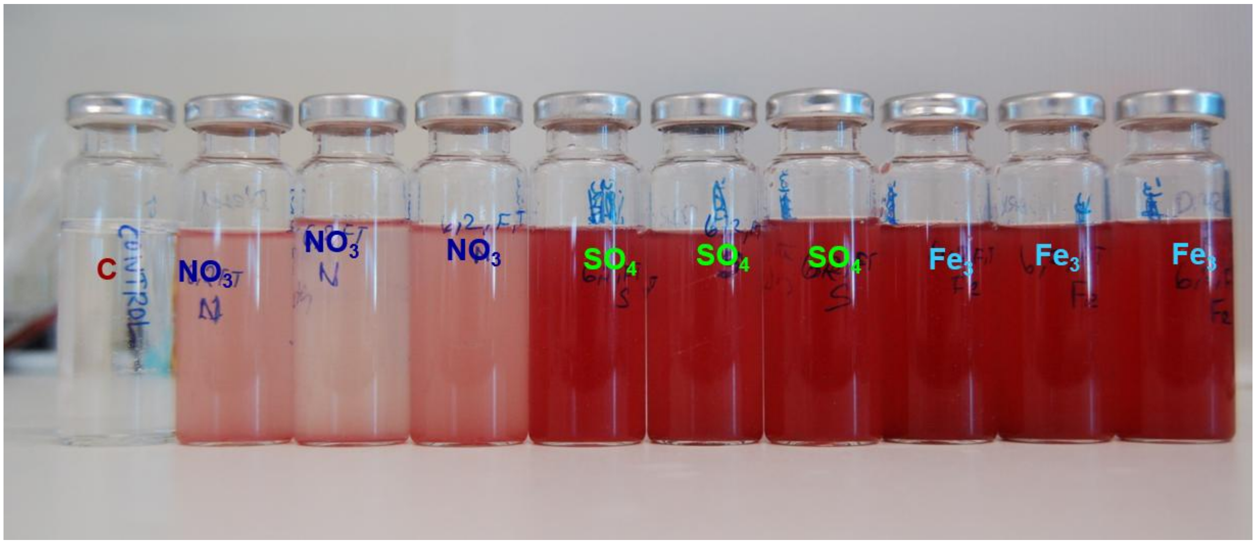

Table. S1. Comparison between exoelectrogenic strains of *Rhodopseudomonas* DX1 and *Rhodopseudomonas* palustris strain RP2

**Particulars**	***Rhodopseudomonas palustris* strain RP2**	***Rhodopseudomonas* DX-1 (Adapted from Xing *et al*. 2008)**
Cell diameter(μm)	0.5-1	0.5-0.8
Cell shape	Rod	Rod
Cyst formation	+	NR
Optimum pH	6.5-7.4	6.5-7.0
Aerobic growth in dark	+	+
Growth factor	p-ABA, pyridoxine HCl, folic acid	p-ABA
Nitrate-reduction	+	-
Sulfate-reduction	+	+
Diesel degradation	+	NR
Formate	-	±
Propionate	+	±
Butyrate	+	±
Methanol	NR	±
Ethanol	-	±
Glutamate	+	+
Gluconate	+	-
Aspartate	+	-
Benzoate	+	+
Glycerol	+	+
Thiosulfate	+	+
Sulfide	+	+

(+): Positive reaction; (-): Negative reaction; (±): Reaction is not stable; (NR): Not reported

Table. S2. Comparison of metabolic genes from the genome of *Rhodopseudomonas palustris* strain RP2

Details	RP2	DX1	ATCC	TIE-1	BisA53	BisB18	BisB5
Nitrate reduction	+	-	-	+	+	+	-
Alkane sulfonate monooxygenase	+	NA	NA	-	-	-	-
Catechol 1,2-dioxygenase	+	NA	NA	+	-	-	-
Gluconate assimilation	+	-	+	-	+	+	+
Aspartate Assimilation	+	-	+	+	-	-	-

(+): Present; (-): Not present; (NA): Not Available

Table. S3. Oligonucleotide primers used in this study

Primers	Sequence(5'-3')	PCR fragment size (bp)	References
ALK1F<br>ALK1R	CATAATAAAGGGCATCACCGT<br>GATTTCATTCTCGAAACTCCAAAC	185	[1]
ALK2F<br>ALK2R	GAGACAAATCGTCTAAAACGTAA<br>TTGTTATTATTCCAACTATGCTC	271	[1]
ALK3F<br>ALK3R	TCGAGCACATCCGCGGCCACCA<br>CCGTAGTGCTCGACGTAGTT	330	[1]
alkBF<br>alkBR	GCGCAAGCTTCCGATTAGCTCAG<br>GCACTCTTTGTGAGAGAATTCAAC	204	[5]
alkB1F<br>alkB1R	AAYACNGCNCAYGARCTNGGNCAYAA<br>GCRTGRTGRTCNGARTGNCGYTG	550	[2]
alkB2F<br>alkB2R	TGGCCGGCTACTCCGATGATCGGAATCTGG<br>CGCGTGGTGATCCGAGTGCCGCTGAAGGTG	870	[2]
alcB1F<br>alcB1R	CGGGGTTCAAGGTCGAGCAT<br>CAGGACCAGGTTGGTGAAGA	434	[2]
alkbF<br>alkbR	CCGGTGTGGTGGTGGATCGG<br>GCGAGCRTGATCATGCT	600	-
alkFGf<br>alkFGr	CACGCAGAGCTCGGCGGTCG<br>GGCTAGGCGAATTCGTATGGTC	500	-
alkMup<br>alkMDn	CGGGGTAAGCATGAATAGCT<br>CGTACAGCTACTTGGTGGAC	496	[4]
DEGF<br>DEGR	CGACCTGATCATGCCATGACCGA<br>TCTAGGTCAGTACACGGTCA	238	[5]
Cy.450<br>palFW<br>palRV	GGCAATTGACGATGCACGGCACCATCG<br>TTTTAATTAAAGGTCATGAGACCCGGACC	-	[6]
R.alkB1F<br>R.alkB1R	ACCCCAGATTGGCGTTCTCC<br>TGCGATCCAACGCTGATGCC	400	This study
alkAF<br>alkAR	TGGGTATGGGACTGGCAAGCGA<br>AGTGAACGGCGCGCAGACAG	192	This study
rub F<br>rub R	GCTGCACCATCTGGAAATCGGCTT<br>ATGAGCGATGCCACCCCCAC	194	This study

References:

1. Kloos, K., Munch, J.C. & Schloter, M. A new method for the detection of alkane-monooxygenase homologous genes (*alk*B) in soils based on PCR-hybridization. *J. Microbiol. Methods* 66, 486-496 (2006).
2. Kohno, T., Sugimoto, Y., Sei, K. & Mori, K. Design of PCR Primers and Gene Probes for General Detection of Alkane-Degrading Bacteria. *Microbes. Environments* 17, 114-121 (2002).
3. Mesarch, M.B., Nakatsu, C.H. & Nies, L. Development of catechol 2, 3-dioxygenase-specific primers for monitoring bioremediation by competitive quantitative PCR. *App. Environ. Microbiol.* 66, 678-683 (2000).
4. Smits, T.H., Röthlisberger, M., Witholt, B. & Van Beilen, J.B. Molecular screening for alkane hydroxylase genes in Gram-negative and Gram-positive strains. *Environ. Microbiol.* 1, 307-317 (1999).
5. Wang, W., Wang, L. & Shao, Z. Diversity and abundance of oil-degrading bacteria and alkane hydroxylase (alkB) genes in the subtropical seawater of Xiamen Island. *Microb. Ecol.* 60, 429-439 (2010).
6. Van Beilen, J.B. et al. Cytochrome P450 alkane hydroxylases of the CYP153 family are common in alkane-degrading eubacteria lacking integral membrane alkane hydroxylases. *Appl. Environ. Microbio*l 72, 59-65 (2006).
